# Update on Obesity and Cardiovascular Risk: From Pathophysiology to Clinical Management

**DOI:** 10.3390/nu16162781

**Published:** 2024-08-20

**Authors:** Giovanna Gallo, Giovambattista Desideri, Carmine Savoia

**Affiliations:** 1 Department of Clinical and Molecular Medicine, Faculty of Medicine and Psychology, Sapienza University of Rome, 00189 Rome, Italy; giovanna.gallo@uniroma1.it; 2 Department of Clinical, Internal Medicine, Anesthesiological and Cardiovascular Sciences, Sapienza University of Rome, Piazzale Aldo Moro 5, 00185 Rome, Italy; giovambattista.desideri@uniroma1.it

**Keywords:** obesity, cardiovascular disease, GLP1-RA

## Abstract

Obesity is an epidemic worldwide. Overweight and multiple obesity-related mechanisms, including dysmetabolic alterations, contribute to cardiovascular deleterious effects. Hence, overweight and obesity have been independently associated with increased cardiovascular risk, whose assessment is crucial for preserving life quality and reducing mortality, and to address appropriate therapeutic strategies in obese patients. Beyond the standard of care in managing overweight and obesity in adults (i.e., diet and physical exercise), several relevant pharmacotherapies have been approved, and several procedures and device types for weight loss have been recommended. In such a contest, medical weight management remains one option for treating excess weight. Most drugs used for obesity reduce appetite and increase satiety and, secondarily, slow gastric emptying to reduce body weight and, therefore, act also to improve metabolic parameters. In this contest, agonists of the glucagon-like peptide-1 receptor (GLP-1RAs) modulate different metabolic pathways associated with glucose metabolism, energy homeostasis, antioxidation, and inflammation. Moreover, this class of drugs has shown efficacy in improving glycemic control, reducing the incidence of cardiovascular events in type 2 diabetic patients, and reducing body weight independently of the presence of diabetes. Recently, in overweight or obese patients with pre-existing cardiovascular disease but without diabetes, the GLP-1RA semaglutide reduced the incidence of cardiovascular and cerebrovascular events and death from cardiovascular causes. Thus, semaglutide has been approved for secondary prevention in obese people with cardiovascular disease. Nevertheless, whether this class of drugs is equally effective for primary prevention in obese people has to be demonstrated. In this review, we will summarize updates on the pathophysiology of obesity, the effects of obesity on cardiovascular risk, the impact of different obesity phenotypes on cardiovascular diseases, and the novelties in the clinical management of obesity for cardiovascular prevention.

## 1. Introduction

Obesity is an epidemic worldwide, accounting for 650 million adults in 2016 and an estimated prevalence of one billion in 2030. In Europe, 60% of inhabitants are either overweight or obese. In developed countries, nearly one-third of the population is predicted to be overweight or obese within the next few years [1,2].

Pathophysiological mechanisms involved in developing obesity and related dysmetabolic conditions are complex, including genetic, neurohormonal, environmental, pharmacological, and psychological factors.

Ectopic fat accumulation and dysregulated adipose tissue represent the main features of obesity and lead to insulin resistance, lipotoxicity, and activation of proinflammatory pathways, which are involved in the progression of vascular and heart damage and increase the risk of cardiovascular events [3].

The impact of obesity on cardiovascular morbidity is enhanced by the coexistence of several comorbidities in obese subjects. These include dysmetabolic conditions such as diabetes, hypertension, dyslipidemia, obstructive sleep apnea hypoventilation syndrome, nonalcoholic fatty liver disease, as well as other diseases including cardiovascular disease (CVD), cancer, chronic kidney disease, neuromuscular and orthopedic disorders with debilitating influence on the quality of life, prognosis, and health-care costs [4]. In this view, it is mandatory in clinical practice to reduce overweight and obesity-related cardiometabolic risk to decrease the burden of CVD [5]. Different pharmacological and non-pharmacological strategies have been proposed to reduce body weight effectively. In such a context, precision nutrition represents an emerging field of interest, consisting of tailored dietary and lifestyle recommendations. Regarding pharmacological strategies, glucagon-like peptide-1 receptor agonists (GLP1-RA) have shown significant results independent of diabetes in reducing body weight and cardiovascular risk [6].

In this narrative review, we will discuss an update on the pathophysiological mechanisms involved in developing obesity and obesity-related metabolic and CVD, the obesity phenotypes and their association with cardiovascular events, and available new pharmacological strategies in reducing body weight and cardiovascular risk.

## 2. Obesity, Inflammation and Adiposopathy

Historically, body mass index (BMI) has been indicated as the main parameter to define obesity (BMI ≥ 30 kg/m^2^). However, this anthropometric measure may be influenced by age, sex, and race/ethnicity. In this view, other parameters have been introduced in clinical practice, such as waist circumference (WC), waist-hip ratio (WHiR), waist-to-height ratio (WHtR), bioimpedance, 3D scanning and dual-energy X-ray absorptiometry (DEXA), with increasing attention to a qualitative rather than to a quantitative assessment of adipose tissue distribution [7,8].

**Figure 1:**
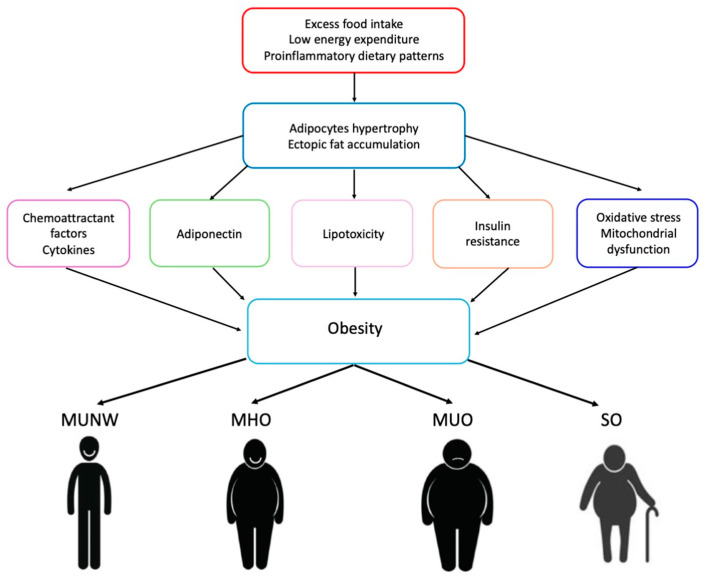
Pathophysiological mechanisms of obesity. MUNW: metabolic unhealthy normal weight; MHO: metabolically healthy overweight/obese; MUO: metabolically unhealthy overweight/obese; SO: sarcopenic obesity.

Pathophysiological mechanisms involved in developing obesity and related metabolic conditions are complex, including genetic, neurohormonal, environmental, pharmacological, and psychological factors. Adipocyte hypertrophy in visceral adipose tissue (VAT), ectopic fat accumulation, and dysregulated adipokine production represent pillar pathophysiological processes in developing obesity and metabolic disorders as well as the progression of adiposopathy [9,10] (Figure 1). The latter condition involves functional changes of adipose tissue, including impaired adipogenesis, adipocyte lipolysis over lipogenesis, increased free fatty acid release, endocrinopathies (hypoadiponectinemia and hyperleptinemia), pathogenic adipose tissue immune responses with a proinflammatory milieu, and pathogenic “crosstalk” between adipose tissue and other organs which ultimately are associated with fat dysfunctional fat accumulation and increased cardiovascular risk.

Obesity is characterized by excess nutrient consumption that contributes to impaired interaction between adipocytes and the immune cells infiltrating the adipose tissue, causing the release of several proinflammatory mediators [11,12].

The effects of nutrients on adipose tissue depend on both quantitative and qualitative aspects. Dietary patterns high in glycemic load and energy density and low in fiber have been suggested to influence body composition, associated with greater fat mass and increased risk of excess adiposity, and to affect innate appetite control with greater energy consumption [13]. Also, polyunsaturated fatty acid consumption has been associated with childhood overweight and obesity. In contrast, a higher protein intake was not associated with a later risk of overweight [13].

Proinflammatory dietary patterns play an essential role in developing adiposopathy and obesity. These diets include low fiber and antioxidant intake, a few antioxidant polyphenols, and immunomodulatory fatty acids, such as eicosapentaenoic fatty acid (EPA) and docosahexaenoic fatty acid (DHA). These diets are also rich in lipoperoxidated vegetable oils, xenobiotics, excessive sodium, and high saturated and trans fats. Thus, proinflammatory diets may promote the accumulation of ceramides and diacylglycerols, as well as impair glucose uptake, PPARγ activity, and insulin signaling, as well as increase cytokine secretion [14].

An increase in monocyte chemoattractant protein-1 (MCP-1), tumor necrosis factor-alpha (TNF-α), interleukin (IL)-6, and leptin levels, and a decrease in adiponectin have been shown to enhance adipogenesis, atherogenesis, fibrosis, and inflammation [15,16].

VAT hypertrophy and inflammatory status are associated with limited oxygen diffusion, leading to mitochondrial dysfunction, reactive oxygen species (ROS) release, and oxidative stress, as well as inducing the activation of HIF-1α [17]. Thus, hypoxic conditions frequently occur in VAT, HIF-1α protein upregulation, influencing angiogenesis, glucose metabolism, cellular stress response, extracellular matrix remodeling, and inflammatory signaling [18].

Adipose tissue is also the primary source of circulating miRNAs in patients with obesity, which enhances autophagy, apoptosis, and inflammatory pathways [19]. Knockout of autophagy-related genes has been shown to improve insulin resistance and endoplasmic reticulum stress and to promote lipid metabolism through the browning of white adipose [20]. Moreover, in obese patients, chronic p53 activation increases M1 macrophage polarization via the NF-κB pathway and enhances the systemic inflammatory response [21].

Adipocytes also release chemerin, a newly characterized chemoattractant that induces the infiltration of innate and adaptive immunity cells and the activation of inflammatory pathways [22,23]. Indeed, inflammation plays a fundamental role in the promotion of obesity-related systemic diseases. Inflammatory mediators induce an M1 macrophage polarization and activate dendritic cells (DC), B, CD4 + T, and CD8 + T cells [22,23,24,25]. Functional overlap between macrophages and adipocytes has been described, since both cell types are activated by lipopolysaccharide and other bacterial products and can secrete cytokines [26]. In addition, pre-adipocytes can transdifferentiate into M1 macrophages. M1 phenotype shifting is associated with the production of IL-1β, IL-6, tumor necrosis factor α (TNFα), and ROS, which further reduce insulin signaling in adipocytes [27,28].

Healthy adipose tissue is characterized by the presence of CD4+ and T-Reg. In contrast, hypertrophic adipose tissue contains CD4+ and CD8+ cytotoxic effector T lymphocytes, which secrete perforin and granzyme B, eliminating damaged cells [29].

The accumulation of VAT is associated with the activation of the Th17 population in the adipose tissue by CD11c+ DC cells, suggesting the importance of innate/adaptive immune cell crosstalk in obesity-associated inflammation [30]. Proinflammatory Th1 CD4+ T cells have been shown in VAT to accelerate obesity and non-alcoholic fatty liver disease [31,32]. Accordingly, it has been hypothesized that VAT could serve as a reservoir of immune cells. Increased levels of circulating immunoglobulin G and A and B cell accumulation have been described in the adipose tissue of obese patients, contributing to insulin resistance [33]. This inflammatory and dysregulated energetic environment is characterized by increased glucose and free fatty acid levels associated with ROS, which activate a post-mitotic cell cycle and a senescent cell program, sustaining a low-grade chronic inflammation [34].

Obesity-associated insulin resistance leads to lipotoxicity and the accumulation of excess fat in non-fat tissues, promoting the activation of pattern recognition receptors (PRR) recognizing pathogen-associated molecular patterns (PAMPs) [35]. PRRs include different types of receptors, such as toll-like receptors (TLRs), nucleotide-binding oligomerization domain-like receptors, C-type lectin receptors, and retinoic acid-inducible gene I-like receptors [36]. TLR4 has been shown to recognize lipopolysaccharides (LPS) of the outer wall of Gram-negative bacteria [37]. In such a context, the role of the mother’s gut microbiota, transferred to the fetus through maternal nutrition, and of maternal nutrition and lifestyle habits are gaining increasing importance due to their epigenetic influence on DNA methylation, histone acetylation, and availability of enzymatic substrates, as well as their impact on early fetal and neonatal microbiome and offspring’s metabolism [38].

Patients with obesity often present increased gut permeability and absorption of bacterial endotoxins due to a predominance of Firmicutes phyla (i.e., Lactobacillus and Faecalibacterium) and Escherichia coli rather than Bacteroidetes [39]. In addition, this gut microbiota composition has been shown to produce fewer levels of short-chain fatty acids (SCFAs), including acetate, propionate, and butyrate, thus generating foam cell formation and favoring the mobilization of lipopolysaccharides, trimethylamine *N*-oxide, and phenylacetyl glutamine (PAGIn) in the general circulation, resulting in intestinal and systemic inflammation [40].

The prevalence of Actinobacteria in human milk has been associated with the adiposity of women before pregnancy and during lactation and has also influenced the offspring’s nutrition status [41]. Maternal hyperglycemia and obesity represent independent risk factors for childhood obesity, being associated with leptin secretion and food intake behaviors [42].

## 3. Obesity and Cardiovascular Diseases

**Figure 2:**
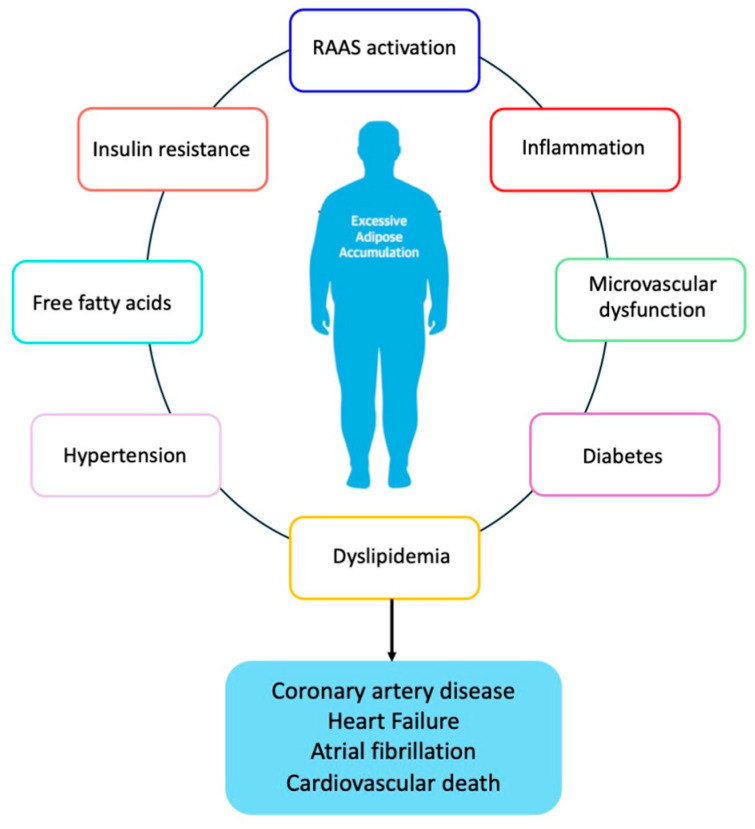
Obesity and development of cardiovascular disease RAAS; renin–angiotensin–aldosterone system.

A large body of evidence has demonstrated the association between obesity and CVD, including atherosclerotic events and heart failure (HF) and indeed, the secretion of growth factors, cytokines, and vasoactive peptides by VAT, neurohormonal dysfunction, insulin resistance and hyperinsulinemia, the activation of the renin–angiotensin system (RAAS) and inflammation induced cardiac fibrosis, cellular death, and microvascular dysfunction [3,43,44]. These conditions are associated with hypertension and other features of metabolic syndrome as well as coronary heart disease and heart failure development (Figure 2).

An association between obesity and hypertension has been described. Different mechanisms have been proposed, including vasoconstriction and renal sodium absorption through the activation of the sympathetic nervous system and the renin–angiotensin–aldosterone system by the VAT-released leptin. Moreover, the increased circulating free fatty acids also result in oxidative stress, inflammation, and metabolic dysregulation [8,45].

Up to 70% of patients with obesity have dyslipidemia, with elevated levels of triglycerides, low-density lipoprotein (LDL) cholesterol, apolipoprotein B, and non-high-density lipoprotein (HDL) cholesterol, as well as with an increase in small dense pro-atherogenic LDL particles [45,46]. A well-established relationship also exists between central/abdominal obesity and diabetes, recognized with the coined term “diabesity.” Moreover, direct vascular dysfunction and obesity-induced metabolic risk factors are associated with coronary atherosclerotic plaque formation. Higher central adiposity at each level of BMI is associated with an increased risk of coronary artery disease and cardiovascular mortality. The degree and duration of obesity (expressed as BMI-years and WC-years) have been shown to predict the risk of CAD [8,45]. Accordingly, favorable changes in body composition have been demonstrated to reduce cardiometabolic risk and morbidity [8,45].

Collagen deposition, fat accumulation, and inflammatory infiltration occur within the vascular wall, leading to progressive arterial thickening [46]. In physiological conditions, perivascular adipose tissue provides mechanical protection and regulates blood vessel tone, secreting adiponectin and angiotensin (1–7) with antithrombotic and vasodilating effects [47,48]. On the other hand, obesity induces a shift to a pro-oxidant and pro-inflammatory environment, leading to endothelial dysfunction, vasoconstriction, vascular stiffness, and atherogenesis [49]. In such a context, the volume of epicardial fat, which is increased in obesity, has been associated with coronary plaque instability, atrial fibrillation, and HFpEF [50]. It has been demonstrated that epicardial fat releases exosomes carrying lipids, proteins, and microRNAs, which are involved in adverse cardiac remodeling [51].

Also, ectopic fat accumulation in the liver, skeletal muscle, and kidney contribute to lipotoxicity, mitochondrial dysfunction, and hypoxia, leading to cytokine and growth factor unbalance and vascular and myocardial damage [52].

Cardiac fibrosis has been described in obese patients, representing a fundamental pathophysiological mechanism in developing cardiac remodeling and dysfunction and heart failure with preserved ejection fraction (HFpEF) [53].

Several pathways are involved in the fibrogenic process, including the cascades of transforming growth factor β (TGF-β), epidermal growth factor (EGF), insulin-like growth factor-1, growth differentiation factor-11, and connective tissue growth factor (CTGF) [54,55,56]. Also, matricellular proteins, including thrombospondins, secreted protein acidic and rich in cysteine (SPARC), and osteopontin, are upregulated in obesity and metabolic syndrome, inducing the deposition of collagen and the proliferation of cardiac fibroblasts [57]. In addition, the increased expression of neprilysin on the surface of mature adipocytes of obese subjects reduces natriuretic peptides, contributing to cardiac inflammation and fibrosis [58].

Obesity has also been associated with an increased risk of cancer as a consequence of metabolic dysfunction [59]. Although the pathophysiological mechanisms should be better clarified, it has been hypothesized that releasing cytokines and chemokines from the excess adipose tissue and insulin resistance might lead to a preneoplastic microenvironment. In addition, obesity-related alterations in the gut microbiome may also contribute to chronic inflammation and a higher cancer risk [59].

Based on available evidence, different phenotypes of obesity have been described including metabolic unhealthy normal weight (MUNW), metabolically healthy overweight/obese (MHO), metabolically unhealthy overweight/obese (MUO), and sarcopenic obesity (SO) [60] (Figure 1).

The critical factor characterizing MHO and MUO, representing the most common phenotypes, is alterations in body fat distribution [61]. MUO is characterized by increased WC, reduced subcutaneous fat, and a shift toward a visceral and pro-inflammatory hypertrophic adipose tissue distribution, with a dysfunctional deposition in the liver and skeletal muscle [62]. Ectopic fat and adipose tissue dysfunction lead to diabetogenic and atherogenic secretion patterns, contributing to MUO development and related CVD [63]. On the other hand, MHO is characterized by high BMI with a favorable lipid profile, low levels of inflammatory cytokines, and preserved insulin sensitivity [64], with no evidence of other dysmetabolic disorders (i.e., type 2 diabetes, hypertension, dyslipidemia) and atherosclerotic cardiovascular disease, as well as with a low rate of CVD development among obese patients [65]. To better classify MHO, Lavie and colleagues proposed the following diagnostic criteria: serum triglycerides ≤ 150 mg/dL), HDL-cholesterol > 40 mg/dL in men or >50 mg/dL in women, systolic blood pressure ≤ 130 mmHg, diastolic blood pressure ≤ 85 mmHg, no antihypertensive treatment as an alternative indicator, fasting blood glucose ≤ 100 mg/dL, and no drug treatment with glucose-lowering agents [66]. The risk of diabetes, CVD, and all-cause mortality is more significant in the MUO than in the MHO phenotype as well as the adverse outcomes are directly related to the number and severity of metabolic abnormalities [67]. This phenotype is more common among young, physically active females who are at lesser risk of CVD development [68]. It has been hypothesized that the differences between MUO and MHO might derive from different biological properties of adipocytes regarding oxidative stress metabolites, ceramides, sphingolipids, and amino-acid metabolism from visceral and subcutaneous adipose tissue rather than the location of the fat depot [69]. Defective adipogenesis has been proposed to play a pivotal role in developing adipose tissue dysfunction, systemic insulin resistance, inflammation, and related metabolic disorders [70]. Clinical studies have suggested that MHO is a transient phenotype in about one-third of obese individuals and that those who maintained an MHO phenotype had a risk of diabetes and CVD comparable to that of metabolically healthy, normal-weight subjects [71]. Longitudinal studies showed that up to 50% of subjects with MHO may convert to MUO within 20 years as a consequence of a decline in insulin sensitivity with increased fasting blood glucose and of the detrimental effect of prolonged excess adiposity [72]. The risk of transitioning from MHO to MUO is higher in subjects with high BMI, constant weight gain during the observational period, older age, and the presence of hepatic steatosis [73]. Despite the low estimated cardiovascular risk in MHO, the incidence of cardiovascular events, including coronary heart disease, cerebrovascular disease, HF, respiratory diseases, and all-cause mortality, is significantly higher in MHO individuals compared to age and sex-matched individuals with average weight [74].

Despite normal weight, the MUNW phenotype is characterized by increased visceral adiposity with an unbalanced fat/lean mass ratio. Changes in anthropometric parameters are also present. These include increased WC, WHiR, and WHtR [75]. MUNW is typically seen in older and sedentary individuals with a very low amount of gluteal-femoral fat mass compared with the visceral one [75]. Although MUNW is associated with an elevated cardiovascular risk [75], this obesity phenotype is often underdiagnosed since not uninvoked criteria are used for the diagnosis of the body fat mass, which ranges from 19 to 32% for men and 29 to 44% for women in different clinical settings [76].

Loss of skeletal muscle due to a sedentary lifestyle, chronic comorbidities, and impairment of VAT, associated with an absolute body fat gain, are the characteristics of the SO phenotype [77]. In such a context, impaired function of the growth differentiation factor myostatin and other myokines, including FNDC5/irisin, fibroblast growth factor 21, and brain-derived neurotrophic factor, has been proposed to contribute to the development of this phenotype, resulting in the inhibition of insulin signaling and lipid oxidation [78]. In addition, obesity and sarcopenia may enhance each other since muscle loss, with consequent reduced mobility, facilitates weight gain, which further worsens exercise capacity in a vicious circle, particularly in the elderly [79]. SO is associated with a poor prognosis due to the increased risk of falls and fractures, hospitalizations, and all-cause mortality [80,81].

All the different described obesity phenotypes have been shown to be related to CVD. Individuals with MHO have been shown to have an increased risk of HF and atrial fibrillation but without significant differences in myocardial infarction (MI), stroke, and cardiovascular death compared to healthy subjects [82].

A direct negative effect of energetic dysmetabolism on cardiac structure, mainly consisting of left ventricle hypertrophy (LVH) and HFpEF, has been reported in MUO and MUNW [82]. Different from age and exercise-related adaptative LVH, this pathological concentric LV remodeling develops in response to increased myocardial stress and arterial afterload and is associated with a change in cardiomyocyte phenotype and an excessive synthesis of extracellular matrix and fibrosis [83]. In addition, LVH represents an arrhythmic substrate with an additional risk of cardiac death triggered by co-factors such as hypoxia, hypercapnia, obstructive sleep apnea, and coronary artery disease [84].

Moreover, in MUNW individuals, a higher incidence of subclinical atherosclerosis, with soft atherosclerotic plaques and an increased risk of plaque rupture and ischemic events, has been reported compared to healthy individuals [85].

A higher incidence of cardiovascular events, including MI, HF, and atrial fibrillation, in subjects with SO compared to those with normal body composition has been described [86]. Other studies demonstrated a poor prognosis after MI in SO patients, characterized by an increased rate of all-cause death, recurrent MI, ischemic stroke, and hospitalization for HF [87]. Indeed, sarcopenic obesity is characterized by several pathological alterations that may be associated with CVD, including increased oxidative stress and mitochondrial dysfunction [88]. The loss of skeletal muscle mass with the accumulation of intramuscular fat and the impairment of contractile function is associated with insulin resistance and metabolic abnormalities, which play a role in developing endothelial dysfunction, vascular remodeling, and cardiovascular events [89].

## 4. Therapeutic Strategies in Obesity Management

Although a primary objective should be the prevention of obesity before its development, integrated treatment initiatives should be encouraged and intensified when this pathology has already occurred [4]. Different pharmacological and non-pharmacological strategies have been proposed to reduce body weight effectively.

Excessive eating habits and proinflammatory dietary patterns should be carefully modulated and tailored to each individual. In particular, a diet low in calories and with nutrients with anti-inflammatory and immunomodulatory properties should be encouraged to reduce VAT accumulation with inflammatory phenotype [14,15]. Therefore, there should be recommendations for food rich in fibers with antioxidant polyphenols—such as eicosapentaenoic fatty acid (EPA) and docosahexaenoic fatty acid (DHA)—food rich in lipoperoxidated vegetable oils and xenobiotics, and low in salt and high saturated and trans fats. Diet suggestions should be associated with regular physical activity, particularly in childhood. The impact of excessive eating habits and physical activity restriction is particularly relevant during childhood, since in this period, the speed of formation of new fat cells is high, their number being three-fold in obese children compared to those with normal weight [90]. During this life period, environmental factors are of great importance. They might influence physiological feedback mechanisms that regulate food intake, consisting of short-term gastrointestinal stimuli and long-term signals from the adipose tissue [91].

In addition, a food intake at unusual times may produce an internal desynchronization between central and peripheral clocks, resulting in impaired secretion of hormones such as insulin, glucagon, corticosterone, leptin, adiponectin, visfatin, chemerin, and lipocalin, which regulate satiation, and appetite [92]. Also, oscillations in the microbiota composition have been described, with circadian rhythms, contributing to reduced hunger during sleep [93]. On this basis, it has been shown that chrono-disruption in food intake is associated with adverse metabolic effects, such as impaired glucose tolerance and diabetes and an increased risk of obesity [94]. In obese women, longer eating time during the day and quicker eating are described [95]. In addition, night eating has been related to increased BMI, with a reduction of equivalent energy expenditure, an increased risk for weight gain, and an impaired glycemic/insulinemic response [96]. Consistently, it has been shown that more intake of calories in the morning rather than in the evening improves glucose tolerance, particularly in diabetic patients [97].

Regarding other strategies to achieve weight loss, in addition to quantitative interventions on food intake and energy restriction, the consumption of low-energy-dense foods and whole-grain-containing diets represents a fundamental tool, increasing fullness, decreasing the intake of saturated fat and regulating metabolic functions and proinflammatory states [98,99]. Common dietetic strategies consist of hypocaloric diets, Mediterranean diets, high-protein diets (to preserve lean muscle mass and enhance satiety), low or moderate carbohydrate diets, low-fat diets, intermittent fasting, or time-restricted diets [87].

An emerging field of interest is represented by precision nutrition, which consists of tailored dietary and lifestyle recommendations based on nutrigenetics, metabolomics, and metagenomics, including anthropometric parameters, clinical biomarkers, genetic testing of single nucleotide polymorphisms, and analysis of gut microbiome [100].

When associated with dietary interventions, moderate to vigorous physical activity contributes to additional long-term weight loss (up to 20%), which may contribute to improve cardiovascular outcomes. In this regard, physical activity accumulated in bouts of at least 10 min duration is inversely associated with BMI and other parameters of body fatness [101].

Bariatric/metabolic surgery (BMS), defined as the procedures inducing loss of body weight throughout the modification of gastrointestinal physiology, still represents an effective and safe tool for patients with BMI ≥ 40 kg/m^2^ or BMI ≥ 35 kg/m^2^ with comorbidities, being associated with a 14–25% body weight reduction and a significant decrease in the risk of hypertension, diabetes, non-alcoholic fatty liver disease, and mortality [100]. Sleeve gastrectomy and Roux-en-Y gastric bypass account for approximately 90% of all operations performed worldwide with mid- and long-term efficacy. Biliopancreatic diversion with duodenal switch, one-anastomosis gastric bypass, and the less invasive adjustable gastric banding represents an alternative approach [100].

Different pharmacological strategies with varying safety profiles have been proposed to control body weight. These anti-obesity drugs improve weight and metabolic parameters, with different potency and effects. Nevertheless, the currently available data do not support unique and conclusive evidence for reducing hard cardiovascular outcomes. The first drugs consisted of sympathomimetic and serotonin-releasing drugs aimed to suppress appetite and increase energy expenditure, such as phentermine, sibutramine, and dexfenfluramine [102]. However, due to the release of norepinephrine, these agents were associated with an increased risk of cardiovascular adverse effects such as tachycardia, dyspnea, dysrhythmias, MI, and sudden cardiac death. Thus, their use has been progressively abandoned [102]. Other pharmacological agents with limited use in clinical practice are mazindol, which exerts an anorexigenic effect through a central action in the hypothalamus, and orlistat (tetrahydrolipstatin), which reversibly inhibits gastric and pancreatic lipases, avoiding the absorption of free fatty acids [102].

In the last few years, other effective pharmacological strategies with a better efficacy/safety profile have been introduced in clinical practice (Table 1).

The association of bupropion, used for addiction to opioids and alcohol, with naltrexone, used for the treatment of depression and nicotine addiction, has been shown to reduce body weight through the action of hypothalamic nucleus arcuatus and on the dopaminergic mesolimbic system, respectively stimulating pro-opiomelanocortin (POMC) neurons and blocking the negative feedback of β-endorphins [103].

**Figure 3:**
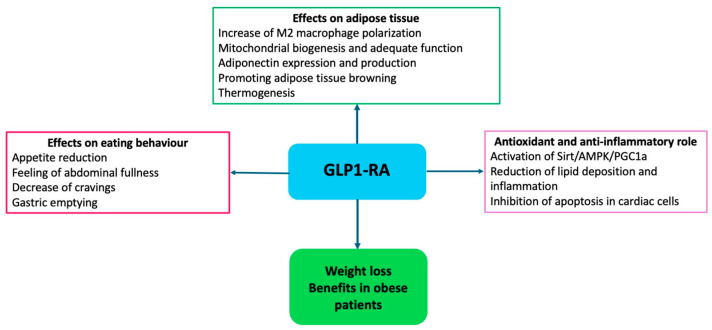
Mechanisms of action of GLP1-RA in obesity.

More recently, GLP1-RA has shown significant results independently from diabetes, stimulating insulin secretion and glucose-lowering, slowing gastric emptying, and increasing hypothalamic sense of satiety through stimulating POMC neurons [104,105] (Figure 3).

Furthermore, GLP1-RA modulates lipid metabolism in white and brown adipose tissues, promoting brown adipose tissue thermogenesis and white adipose tissue browning [106,107]. The activation of the AMPK pathway in brown adipose tissue triggers lipolysis through the release of free fatty acids and the upregulation of uncoupling protein 1 (UCP1), which generates thermogenesis through the release of electrons during oxidative phosphorylation in the inner mitochondrial membrane [108]. Different studies have demonstrated that GLP1-RA decreases ROS production in endothelial cells and cardiomyocytes and reduces the expression of adhesion molecules, such as monocyte chemotactic protein-1 (MCP-1), E-selectin, intercellular adhesion molecule-1 (ICAM-1) and vascular cell adhesion molecule-1 (VCAM-1), and the accumulation of inflammatory cells, as well as inducing the expression of NO synthase-3 (NOS-3) leading to vasorelaxation and slowing the progression of atherosclerotic plaques [108].

In addition, ectopic adipose tissue depots have been reduced by GLP1-RA, which contributes to the favorable effects on the vascular endothelium and myocardium [109]. In the SCALE Obesity and Prediabetes study, liraglutide produced a body weight loss of 7.8 kg, which was maintained for three years and was associated with the improvement of metabolic profile and blood pressure control and with an 80% reduction of the risk of diabetes [110]. The STEP (Semaglutide Treatment Effect in People with Obesity) study and its sub-analyses showed that treatment with semaglutide produced a 5% reduction of body weight in >90% of subjects and a 20% reduction in about 35% [111]. In the STEP-HF study, 529 obese patients with HFpEF were randomized to receive once-weekly semaglutide (2.4 mg) or placebo for 52 weeks to investigate the effects on body weight and quality of life evaluated with the Kansas City Cardiomyopathy Questionnaire clinical summary score (KCCQ-CSS) [111]. Semaglutide produced a more significant improvement of KCCQ-CSS (+7.8 points), a greater reduction in body weight of 10.7%, and a 20.3 m improvement of the 6-min walking test compared to the placebo. No significant differences were reported in the risk of adverse events [112].

Besides their effects on weight reduction and glycemic control, GLP1-RA has been shown to reduce cardiovascular outcomes in obese subjects independently from diabetes. More recently, the SELECT (Semaglutide Effects on Cardiovascular Outcomes in People with Overweight or Obesity) study investigated the effects of semaglutide on the incidence of major adverse cardiovascular events in participants without diabetes and with established CVD, including prior MI, prior stroke, or symptomatic peripheral arterial disease (PAD), and overweight or obesity [113]. The study included 17,604 patients randomly assigned (1:1) to receive once-weekly subcutaneous semaglutide 2.4 mg or placebo [114]. The main inclusion criteria were age ≥  45 years, BMI ≥  27 kg/m^2^, prior MI, stroke, or symptomatic PAD. Exclusion criteria were a history of diabetes mellitus, treatment with glucose-lowering agents within the past 90 days, and HF in New York Heart Association (NYHA) class IV. The primary efficacy endpoint was a composite of death from cardiovascular causes, nonfatal MI, or nonfatal stroke, assessed in a time-to-first-event analysis. Confirmatory secondary endpoints, tested in hierarchical order, were cardiovascular death, a composite HF endpoint consisting of cardiovascular death, hospitalizations or urgent medical visits for HF, and death from any cause. After about 40 months of follow-up, a once-weekly dose of subcutaneous semaglutide produced a 20% reduction of the composite outcome of death from cardiovascular causes, nonfatal MI, or nonfatal stroke [113]. The trial was not powered to analyze statistical significance for mortality and other separate secondary endpoints. However, a trend toward reducing these outcomes has been reported [112]. In the semaglutide group, body weight was reduced by 9.4%, with a steady state reached at ten weeks and maintained throughout the follow-up period. In addition, semaglutide was associated with the reduction of systolic blood pressure, incidence of diabetes, LDL-cholesterol, and C-reactive protein. The effects of semaglutide were independent of the baseline BMI range and were also observed in overweight patients with a BMI < 30 kg/m^2^ [113,114,115].

In this regard, soluble guanylate cyclase stimulators, by improving sensitivity to NO and promoting the cGMP-PKG pathway, might represent a future potential synergistic therapeutic strategy that enhances the anti-fibrotic, anti-inflammatory, and anti-proliferative properties of GLP1-RA [116]. This association may exert favorable effects, particularly on cardiovascular remodeling in obese individuals.

Retatrutide is a novel triple agonist of the glucose-dependent insulinotropic polypeptide, glucagon-like peptide 1, and glucagon receptors. A phase 2 study investigated the effects of retatrutide in 338 obese subjects, showing a body weight reduction of up to 17.5% at 24 weeks and 24.2% at 48 weeks [117].

Tirzepatide is a once-weekly subcutaneous injectable peptide engineered from the native GIP sequence, with agonist activity at both the GIP and GLP-1 receptors, and it has been approved for type 2 diabetes. Tirzepatide also induces a delay of gastric emptying, reduces fasting and postprandial glucose concentration, and decreases food intake, with sustained weight reduction in obese adults [118]. In the ongoing SURPASS-CVOT (comparison of tirzepatide and dulaglutide on major adverse cardiovascular events in participants with type 2 diabetes and atherosclerotic cardiovascular disease), 13,299 diabetic subjects with established atherosclerotic CVD were randomized to receive tirzepatide, a once-weekly GIP/GLP-1 receptor agonist, and dulaglutide, a GLP1-RA, to investigate cardiovascular safety and efficacy [119].

Finally, taking into account the abnormal distribution of adipose tissue in obese patients and the different phenotypes, drug metabolism and elimination may be significantly impacted. Therefore, dose adjustments could be required [120]. This aspect may contribute to the failure of therapeutic strategies and the need to switch an agent to another or prescribe combination treatments to maintain the weight loss rate. Next-generation multi-omics might provide novel targets and predict the success of each therapeutic approach.

## 5. Conclusions

Obesity is a chronic disease with a heavy burden of metabolic and cardiovascular sequelae. Proinflammatory dietary patterns play an essential role in developing adiposopathy and obesity and promote insulin resistance and cytokine secretion, leading to vascular and cardiac damage. In this view, structured pharmacological and non-pharmacological interventions are needed to reduce the risk of cardiovascular events and improve quality of life. Different pharmacological strategies have been introduced to reduce body weight and control cardiometabolic sequelae. Notably, GLP1-RA has been shown to decrease body weight, improve glycemic control, and reduce cardiovascular outcomes, at least in secondary prevention, in obese subjects independently from diabetes. Thus, this class of drugs represents a new effective tool whose prescription should be implemented in clinical practice for cardiovascular protection. Different obesity phenotypes have been described, and further studies are needed to investigate the safety and efficacy profiles of available therapeutic tools in each clinical scenario, particularly for primary prevention.

## Figures and Tables

**Table 1:** Modern pharmacological treatment of obesity.

Pharmacological Class	Mechanism of Action	Effects
Bupropion/naltrexone	Action on hypothalamic nucleus arcuatus and dopaminergic mesolimbic system.	Body weight reduction up to 5%.
GLP1-RA	Stimulation of insulin secretion and glucose-lowering; slowing gastric emptying and increasing hypothalamic sense of satiety through stimulating POMC neurons.	Greater reduction in body weight (+10.7%) compared to placebo. Reduction of the composite outcome of death from cardiovascular causes, nonfatal MI, or nonfatal stroke (−20%) in secondary prevention.
Tirzepatide	Stimulation of first- and second-phase insulin secretion and reduction of glucagon levels, both in a glucose-dependent manner; it also induces a delay of gastric emptying, reduces fasting and postprandial glucose concentration, and decreases food intake.	Sustained weight reduction in obese adults (up to 24%).
Retatrutide	Novel triple agonist of the glucose-dependent insulinotropic polypeptide, glucagon-like peptide 1 and, glucagon receptors.	Body weight reduction up to 17.5% at 24 weeks and to 24.2% at 48 weeks.
